# Autochthonous Leprosy without Armadillo Exposure, Eastern United States

**DOI:** 10.3201/eid2311.171145

**Published:** 2017-11

**Authors:** Tina Rendini, William Levis

**Affiliations:** Bellevue Hospital Center, New York, New York, USA

**Keywords:** immigration, armadillo, Mycobacterium leprae, tuberculosis and other mycobacteria, leprosy, New York, United States, bacteria, zoonoses, Georgia, Florida

## Abstract

Autochthonous leprosy has been reported in New York City, where there are no wild armadillos. Recent autochthonous cases also have been reported in Georgia and Florida and blamed on armadillos, including cases with no known armadillo exposure. International migration needs to be considered as a cause of autochthonous leprosy.

In 1982, we reported that leprosy in New York City occurred exclusively among foreign-born persons (*1*). In 1991, Mastro et al. reported that leprosy was an epidemic phenomenon without secondary transmission (*2*). In 2000, however, the first autochthonous cases of leprosy in New York City were reported (*3*), and 2 additional autochthonous cases subsequently were reported (*4*,*5*). Autochthonous leprosy has been reported in the eastern United States in Georgia (*6*) and central Florida (*7*); transmission was blamed on armadillos, even though most of these case-patients had no history of exposure to armadillos, and armadillos east of the Mississippi River rarely have leprosy (*8*).

Although the transmission of leprosy is poorly understood, international migration of persons with leprosy is a more likely scenario for autochthonous transmission than contact with armadillos, especially if a case-patient has no history of armadillo exposure. Ramos et al. linked an increase in autochthonous leprosy in Spain to a 5-fold increase in migration from countries where leprosy is prevalent (*9*). There are no wild armadillos in New York City. Autochthonous cases of leprosy reported from the eastern United States should not be assumed to be from armadillos. Physicians throughout the United States need to be aware that leprosy can occur in native-born Americans and that delayed diagnosis, which occurs frequently, can result in unacceptable deformities.

Leprosy most commonly is characterized by an infiltrative dermopathy, which dermatologists and many physicians know is an indication for skin biopsy. Many otherwise highly trained physicians are not aware of this indication for a skin biopsy, which is required to diagnose leprosy. This indication is routinely taught in dermatology clinics, but leprosy is common enough in the United States that it should be incorporated into the core curricula of medical schools. Leprosy also can be characterized by fever and arthritis simulating lupus erythematosus, rheumatoid arthritis, or antiphospholipid syndrome because autoantibodies occur in type II reaction known as erythema nodosum leprosum. Physicians should order a Fite stain on the skin biopsy specimen because *Mycobacterium leprae* is sensitive to the alcohol decolorizing step; if only a routine acid-fast stain (Ziehl-Neelsen) is ordered, the diagnosis is often missed (*10*).
